# Taming the fire—transcription factors for redox control in animals and plants

**DOI:** 10.1007/s00709-024-01948-9

**Published:** 2024-04-06

**Authors:** Peter Nick

**Affiliations:** Joseph Gottlieb Kölreuter Institute for Plant Sciences, Karlsruhe Institute of Technology, Karlsruhe, Germany

The first cell was probably born in a world without oxygen, but the innovation of oxygenic photosynthesis more than 2700 million years ago (des Marais 2000) was a breakthrough, because it allowed to exploit carbohydrates more than an order of magnitude more efficiently as compared to anaerobic metabolism (Dismukes et al. 2001). The control over oxygen as acceptor for free electrons was a hallmark for life on our planet, comparable in impact to the control over fire for humanity. Power always comes with a price to be paid: Both, oxygen and fire, are extremely dangerous and can, if remaining untamed, lead to devastating consequences. In case of oxygen, it is the accumulation of incompletely reduced intermediates, the so-called Reactive Oxygen Species that can attack almost any essential molecule in a cell to still their hunger for free electrons.

Control of redox homeostasis is, therefore, mandatory for the survival of all cells, no matter, which life form they constitute. Three contributions to the current issue address the role of transcription factors for this central ability of aerobic life.

Oxidative stress can result not only from excess of oxygen, but also from hypoxia, because the respiratory chain (as well as photosynthetic electron transport) requires a balance of electron donors and acceptors and a controlled steady-state level of electrons in-between. The contribution by Huang et al. (2024) to the current issue is addressing the role of hypoxia in cancer. Blood cancers often come with hypoxia in the bone marrow stromal cells. As adaptation, these cells accumulate a transcription factor, hypoxia-inducible factor-1α, whose function is to orchestrate developmental responses to improve oxygen supply including the formation of new blood vessels. One of the targets is interleukin 8, activating inflammatory responses, supporting tumour progression. The authors provide novel insight into this phenomenon. Subjecting a bone marrow stromal cell line, they can show that hypoxia stimulates expression and secretion of interleukin 8. This response is eliminated in a cell line, where hypoxia-inducible factor-1α is knocked out. Using a dual luciferase promoter-reporter assay, they can show that the transcription factor indeed binds to the promoter of interleukin 8 and activates transcription. Thus, therapeutic interventions targeted to intercept the accumulation of hypoxia-inducible factor-1α, for instance by changing oxygen supply in the bone marrow, should be able to support chemotherapy against the tumour cells themselves.

A link between perturbed redox homeostasis and transcription factors regulating immune responses is also reported for the Huanglongbing disease currently devastating the global Citrus industry. This link emerged from a transcriptomic study by Liu et al. (2024) in the current issue. The causative pathogen, *Candidatus Liberibacter asiaticus*, penetrates into the root system of Citrus trees and hijacks the immune response there. Plant immunity is innate and composed of two layers, a broad-band basal immunity deployed against necrotrophic pathogens, where the challenged cell defends itself by accumulation of anti-microbial compounds, by sealing the penetration site by callose plugs, and by secretion of enzymes that attack the cell wall of the microbes. A second tier is more special and targets biotrophic pathogens—this type of pathogens can silence basal immunity and turn the attacked cell into a zombie, feeding the intruder with sugars. The most efficient strategy against such biotrophic pathogens is to commit suicide, the so-called Hypersensitive Response, which will kill the attacker by the content of the vacuole and, thus, protect the neighbouring tissues. *Candidatus Liberibacter asiaticus* has now invented a devilish strategy—by (still unknown) signals, it can pretend a biotrophic attack (although, in fact, it is necrotrophic) and deploy a Hypersensitive Response of Citrus cells. It just has to stand by and wait, until the suicide is completed and can then devour the corpse. The signalling required to launch Hypersensitive Responses involves activation of apoplastic oxidative burst through a membrane-bound NADPH oxidase termed Respiratory burst oxidase Homologue. Given the decisive impact of this burst, it is of no surprise that this burst is tightly regulated. Growth stimulators such as auxin act as negative regulators. The jasmonate pathway is activated in basal immunity, but is silenced when defence is channelled towards the cell-death-related form of immunity. Comparing the transcriptome of mock-inoculated versus pathogen-inoculated Citrus, the authors find a strong down-regulation of specific *AUX/IAA* genes, transcriptional repressors of auxin-dependent gene expression. This is a paradox, because, at the same time, YUCCA, a key gene for auxin biosynthesis, is strongly upregulated. Since auxin and jasmonate signalling compete for common signalling compounds (for review, see Nick 2006), jasmonate signalling (which would be able to mitigate the inappropriate Hypersensitive Response) should be off. In fact, although the genes for jasmonate biosynthesis are strongly stimulated, while jasmonate catabolism is turned off, the expected strong increase in jasmonate steady-state levels should come with a strong stimulation of *JAZ* genes transcriptional regulators of jasmonate-dependent transcription. However, the *JAZ* genes remain silent. Thus, the bacterium can manipulate the phytohormonal machinery in a way that they quell jasmonate signalling by de-regulation of the auxin pathway, while at the same time upregulating the machinery of oxidative burst.

Can the knowledge about transcription factors involved in the regulation of redox homeostasis be developed into application, for instance by increasing stress resilience of crop plants? This is exactly the topic of the contribution by Samota et al. (2024). They try to increase stress resilience of rice, the most important staple crop on this planet, by seed priming. Here, seeds are exposed to a mild stress to activate resilience for more severe stresses faced later. The authors use methyl jasmonate, a systemic stress signal in plants in two rice varieties that differ with respect to drought tolerance. They can demonstrate that this treatment stimulates redox homeostasis by inducing the expression of antioxidant enzymes. The key is the degradation of JAZ proteins in response to the priming, which activates a transcriptional cascade through a couple of transcriptional activators including the Drought-Response Element Binding (DREB) factors that orchestrate the accumulation of osmoprotectants, but also the transcription of enzymes that detoxify Reactive Oxygen Species formed in consequence of drought stress.

Despite the evolutionary distance between animals and plants, they have to cope efficiently with oxidative challenges as a by-product of any type of stress. Where are the commonalities to be sought here? Certainly not on the level of transcription factors. There is no homology between hypoxia-inducible factor-1α, the AUX/IAA factors, and the JAZ or DREB factors beyond the fact that they can bind to specific signatures in the promoters of their target genes. However, the activation of these factors must be achieved by upstream signalling, and on this tier, evolutionarily ancient factors might be hidden. For instance, Respiratory burst oxidase Homologues as central input for stress signalling in plants are homologues of a subunit from oxidases by which mammalian phagocytes attack intruders (Suzuki et al. 2011), which points to conserved elements that have been retained during evolution and just integrated into novel functional contexts. Integration into context is also needed on the level of scientific understanding—we need to link cell biology with evolutionary viewpoints to extract sense from the complex empirical record.
